# Supplementary material for: Identification of miRNAs Mediating Seed Storability of Maize during Germination Stage by High-Throughput Sequencing, Transcriptome and Degradome Sequencing
Source: Int J Mol Sci. 2022 Oct 15;23(20):12339. doi: 10.3390/ijms232012339 (PMC9604548; doi:10.3390/ijms232012339)
Supplement: Supplementary file 1 [file ijms-23-12339-s001.zip › Supplementary Figure S1.pdf]

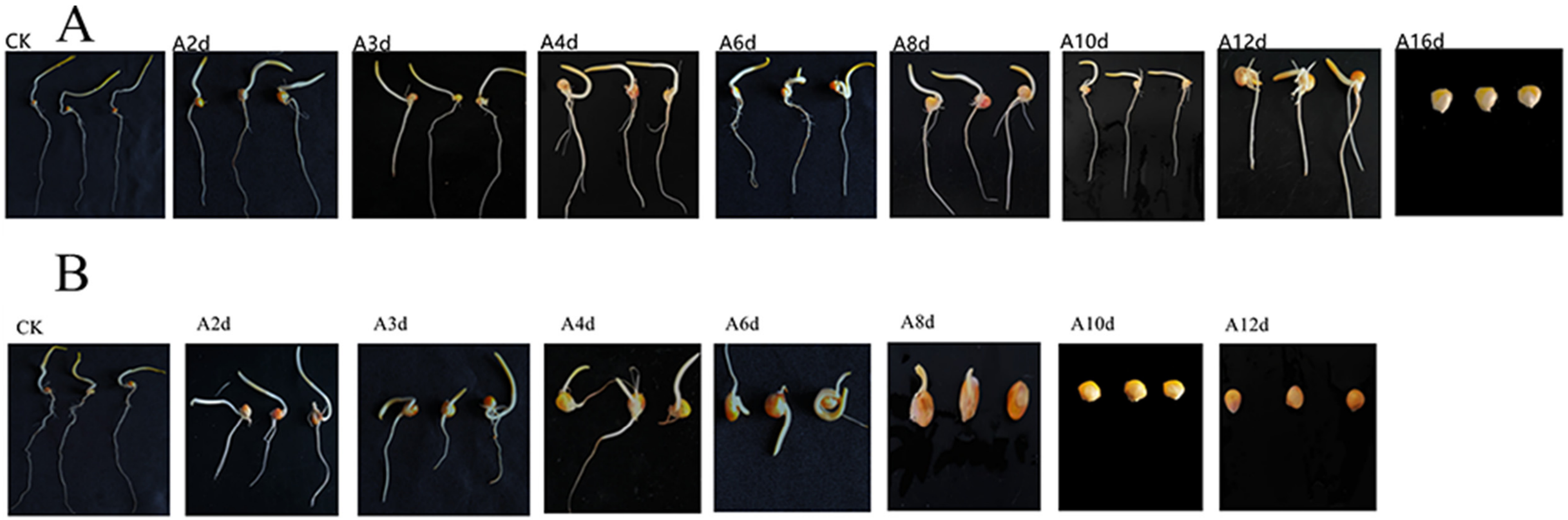

Supplementary Figure S1. Germination of maize inbred lines Dong 156 and Dong 237 under different artificial aging time. (A) the germination of maize inbred line Dong156 under different artificial aging times; (B) the germination of maize inbred line Dong237 under different artificial aging time. CK, A2d, A3d, A4d, A6d, A8d, A10d, A12d and A16d indicate that there is no artificial aging, and the artificial aging is 2d, 3d, 4d, 6d, 8d, 10d, 12d and 16d, respectively.
