# Supplementary material for: Identification of miRNAs Mediating Seed Storability of Maize during Germination Stage by High-Throughput Sequencing, Transcriptome and Degradome Sequencing
Source: Int J Mol Sci. 2022 Oct 15;23(20):12339. doi: 10.3390/ijms232012339 (PMC9604548; doi:10.3390/ijms232012339)
Supplement: Supplementary file 1 [file ijms-23-12339-s001.zip › Supplementary Figure S2.pdf]

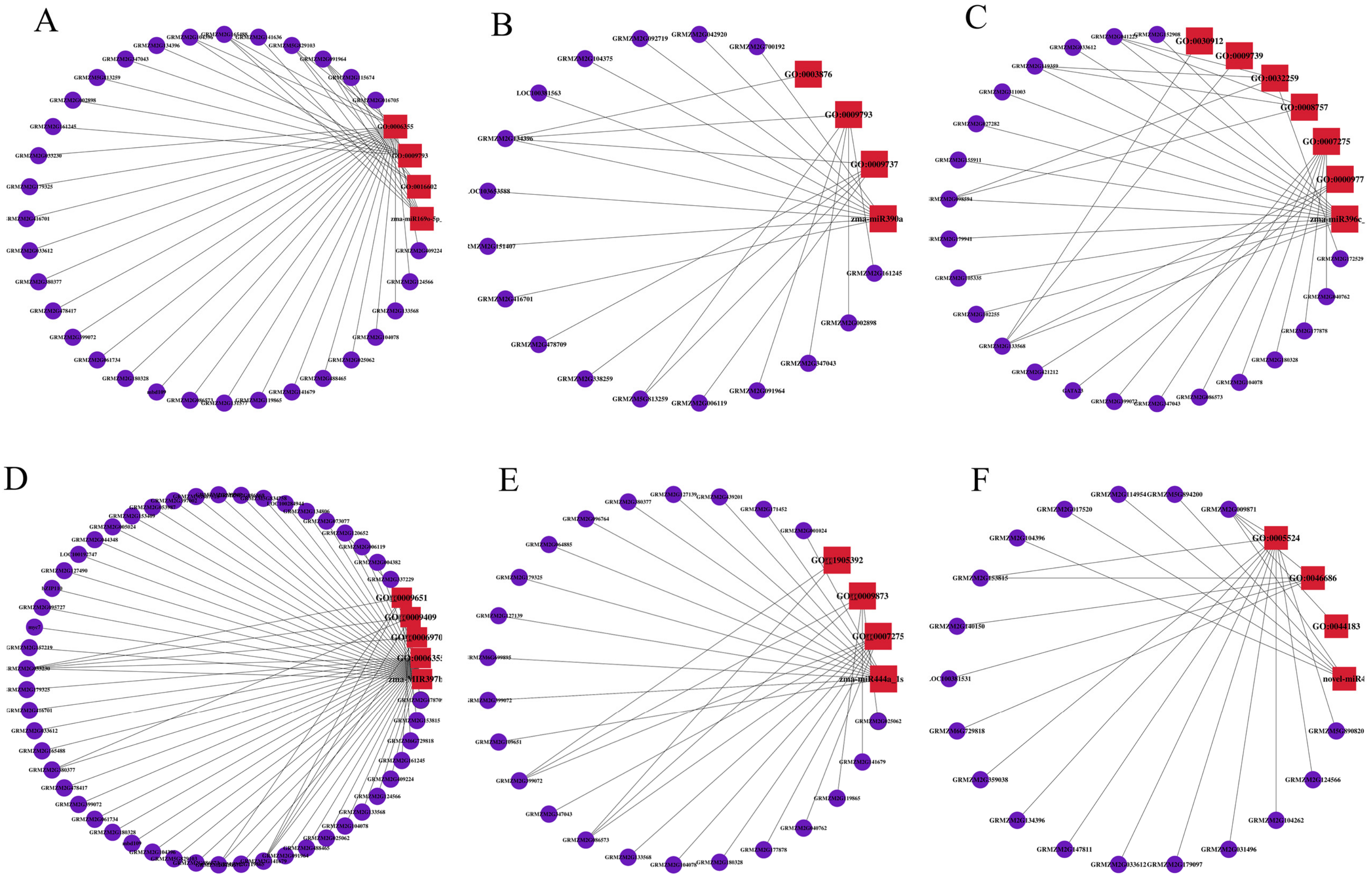
A
B
GO:0009793
GO:0009737
zma-miR390a
C
GO:0030912
GO:0009739
GO:0032259
GO:0008757
GO:0007275
GO:0000977
D
E
F
GO:0005524
GO:0044183

Supplementary Figure S2. miRNA-mRNA network related to GO terms of target genes.

Note: (A) zma-miR169o-5p_ R-1-*GRMZM2G165488* and related GO terms "GO: 0006355, GO: 0009793 and GO: 0016602" corresponding DEGs, (B) zma-miR390a-5p-*GRMZM2G134396* and related GO terms " GO : 0009737, GO:0009793 and GO:0003876" corresponding DEGs, (C) zma-miR396c_ L-1-*GRMZM2G098594*/*GRMZM2G105335*/*GRMZM133568* and related GO terms "GO: 0000977, GO: 007275, GO: 008757, GO-0032259" and other corresponding DEGs. (D) zma-miR397b-p5-*GRMZM2G033230* and related GO terms"GO:0006355，GO：0006970，GO：0009409 and GO：0009651 "corresponding DEGs, (E) zma-miR444a_1ss12TC-*GRMZM2G399072* and related GO terms"GO：0007275,GO：0009873 and GO：1905392"corresponding DEGs, (F) novel-miR4-*GRMZM2G009871* and related GO terms"GO:0046686 and GO:0005524" corresponding DEGs.
