# Supplementary material for: Identification of miRNAs Mediating Seed Storability of Maize during Germination Stage by High-Throughput Sequencing, Transcriptome and Degradome Sequencing
Source: Int J Mol Sci. 2022 Oct 15;23(20):12339. doi: 10.3390/ijms232012339 (PMC9604548; doi:10.3390/ijms232012339)
Supplement: Supplementary file 1 [file ijms-23-12339-s001.zip › Supplementary Figure S3.pdf]

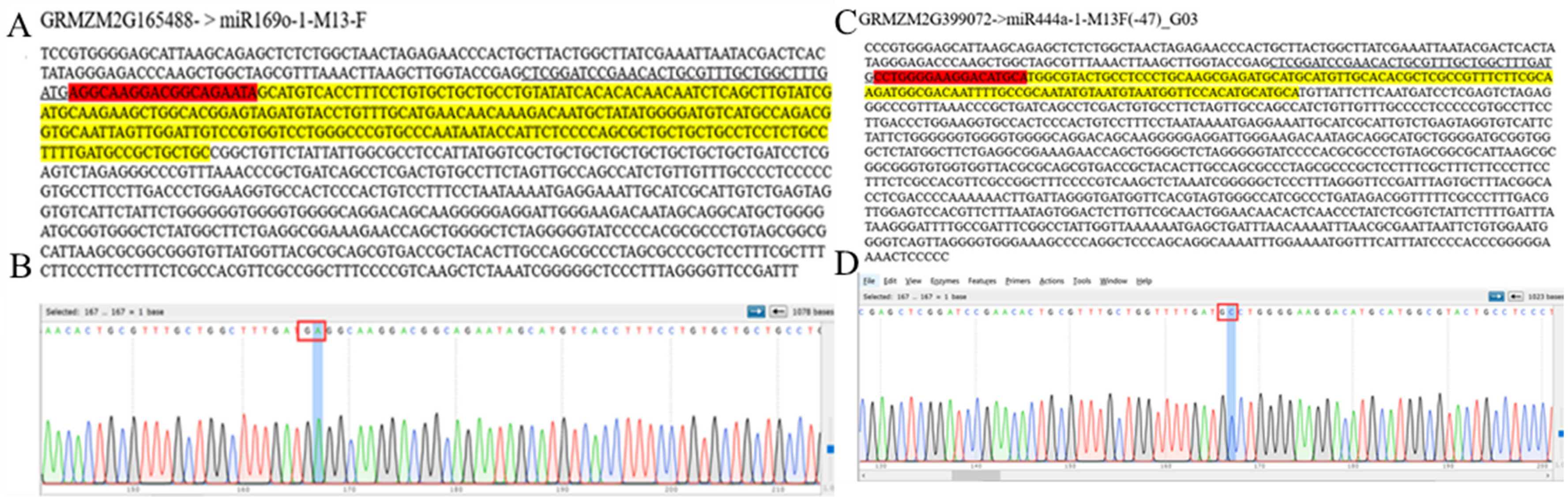

Supplementary Figure S3. miRNA predicts binding site and cleavage site sequence peak map. (A) zma-miR169o-5p_ R-1 predicted binding site, the red part indicates the predicted microRNA binding site, the yellow part indicates the target gene sequence, Underlined is the race connector sequence. (B) zma-miR169o-5p_ R-1 cleavage site sequencing peak map. (C)zma-miR444a_ 1ss12tc predicted binding site, the red part indicates the predicted microRNA binding site, the yellow part indicates the target gene sequence, Underlined is the race connector sequence. (D) zma-miR444a_ 1ss12tc cleavage site sequencing peak map.
