# Supplementary material for: Identification of miRNAs Mediating Seed Storability of Maize during Germination Stage by High-Throughput Sequencing, Transcriptome and Degradome Sequencing
Source: Int J Mol Sci. 2022 Oct 15;23(20):12339. doi: 10.3390/ijms232012339 (PMC9604548; doi:10.3390/ijms232012339)
Supplement: Supplementary file 1 [file ijms-23-12339-s001.zip › Supplementary Table S1.pdf]

Supplementary Table S1 Reading of each microRNA measured by small RNA sequencing data

	AA156_24h1			AA156_24h2			AA156_24h3			AA237_24h1			AA237_24h2			AA237_24h3		
lib	Total	% of Total	uniq	Total	% of Total	uniq	Total	% of Total	uniq	Total	% of Total	uniq	Total	% of Total	uniq	Total	% of Total	uniq
Raw reads	13445996	100	2042901	13721081	100	2128848	14067318	100	2711172	13826716	100	2677869	14163904	100	2101323	13979661	100	1665752
3ADT&length filter	7935369	59.02	667420	8014783	58.41	776875	5612423	39.9	643028	5753479	41.61	547171	9743645	68.79	598996	10843180	77.56	676555
Junk reads	6193	0.05	3381	11817	0.09	5535	17832	0.13	9338	13120	0.09	6784	10166	0.07	5568	7896	0.06	3966
Clean reads	5504434	40.94	664039	5694481	41.5	771340	8437063	59.98	633690	8060117	58.29	540387	4410093	31.14	593428	3128585	22.38	672589
Rfam	447970	3.33	6540	539192	3.93	8477	609320	4.33	8139	852887	6.17	10125	376591	2.66	8114	337046	2.41	7660
mRNA	1759825	13.09	20718	1770754	12.91	22873	2964235	21.07	31029	1885747	13.64	30012	800719	5.65	17912	608489	4.35	13091
Repeats	5015	0.04	172	5615	0.04	198	3068	0.02	158	7879	0.06	218	6464	0.05	164	6857	0.05	139
valid reads	3414551	25.39	1346081	3525350	25.69	1316760	5080938	36.12	2021241	5524271	39.95	2085662	3330512	23.51	1472270	2266188	16.21	965942
