# Supplementary material for: Identification of miRNAs Mediating Seed Storability of Maize during Germination Stage by High-Throughput Sequencing, Transcriptome and Degradome Sequencing
Source: Int J Mol Sci. 2022 Oct 15;23(20):12339. doi: 10.3390/ijms232012339 (PMC9604548; doi:10.3390/ijms232012339)
Supplement: Supplementary file 1 [file ijms-23-12339-s001.zip › Supplementary Table S2.pdf]

Supplementary Table S2 A total of 20 putative miRNAs were differentially expressed

miR_name	miR_seq	up/down	fold_change(AA156_24h(mean)/AA237_24h(mean))	log2(fold_change)	pvalue(t_test)	AA237_24h(mean)	AA156_24h(mean)
PC-5p-8044_611	TAGGTCTTGTTTGGCTGCACT	up	2.347774	1.231294	0.0000	7.4	17.5
PC-5p-162391_23	ATCTCCGACGGCTAGTTACGAGGC	up	49.06233	5.616544	0.0012	0.1	4.9
PC-3p-50720_92	ATCTCCGACGGTCTCGTACAGAGC	down	0.009239	-6.75801	0.0024	10.8	0.1
PC-5p-46450_101	TCCAAGTCAATGCATCATTGC	down	0.00378	-8.04753	0.0030	26.5	0.1
PC-3p-87703_49	ATGTCCGACGGCCTCGTACAGGGC	down	0.008986	-6.79805	0.0047	11.1	0.1
PC-3p-77755_56	GATCAACATTATGTGGCCTGGA	up	66.25966	6.050059	0.0097	0.1	6.6
PC-3p-123986_32	AATAGCGCTATTTCCGACGGTACC	down	0.011775	-6.40813	0.0103	8.5	0.1
PC-3p-91946_46	AATCTGACGGAAACGAATAAGGCC	down	0.019525	-5.67855	0.0129	5.1	0.1
PC-3p-47955_98	AACGAGTTAGTAACGTGGGTACCC	down	0.003429	-8.18796	0.0137	29.2	0.1
PC-3p-24381_202	ATCGTATTAAAAACGTCGGTACCC	up	50.06643	5.645772	0.0194	0.1	5.0
PC-5p-26341_186	ATTACTGTGGCAAGCCAACGGCCT	down	0.063961	-3.96667	0.0265	92.5	5.9

PC-5p-58650_78	AGGAGGATAGGTCCCACCCGC	down	0.192434	-2.37757	0.0318	17.6	3.4
PC-5p-58549_78	CTAATCCACATGTATTGAGGTG	down	0.009212	-6.76232	0.0364	10.9	0.1
PC-3p-46024_102	AGCTTATGTCCGACGGCTTTGGCC	up	80.9108	6.33826	0.0368	0.1	8.1
PC-5p-80184_54	GTATCTTCGGTCGTCGAACTT	up	256.4596	8.002588	0.0373	0.1	25.6
PC-5p-71261_62	TGGTCTTGCCACCGTGTCGTCC	down	0.004632	-7.75426	0.0388	21.6	0.1
PC-3p-20927_237	AATCCCCTCTGAATTAGTGTAACC	down	0.003399	-8.20079	0.0431	29.4	0.1
PC-3p-102177_41	AAACCCCTTCGGTATTTGGACTCC	up	66.60729	6.057608	0.0446	0.1	6.7
PC-3p-25167_195	TTATAGTCCCCTCAATTCTCT	up	130.5913	7.028915	0.0456	0.1	13.1
PC-5p-150211_25	TTGCTTGGGAGCTAGGACCCGAGA	down	0.006503	-7.26471	0.0495	15.4	0.1
