# Supplementary material for: Identification of miRNAs Mediating Seed Storability of Maize during Germination Stage by High-Throughput Sequencing, Transcriptome and Degradome Sequencing
Source: Int J Mol Sci. 2022 Oct 15;23(20):12339. doi: 10.3390/ijms232012339 (PMC9604548; doi:10.3390/ijms232012339)
Supplement: Supplementary file 1 [file ijms-23-12339-s001.zip › Supplementary Table S3.pdf]

Supplementary Table S3 Known miRNA and its corresponding target gene information

MiRNA	Transcript	Describtion
zma-miR156a-5p	*GRMZM5G836353*	uncharacterized protein LOC100217292
zma-miR156e-3p _L+1_2ss14CA22TC	*GRMZM2G119865*	ethylene-responsive transcription factor RAP2-9
	GRMZM2G399072	AP2-like ethylene-responsive transcription factor BBM2 isoform X2
zma-miR162-3p_R+1	*GRMZM2G477314*	CF9
	GRMZM2G040762	uncharacterized protein LOC100381417 isoform X1
	GRMZM2G079066	uncharacterized protein LOC100276268
	GRMZM2G040762	uncharacterized protein LOC100381417 isoform X1
zma-miR164a-5p	*GRMZM2G180452*	putative HLH DNA-binding domain superfamily protein
	GRMZM2G168681	farnesyl pyrophosphate synthase
	GRMZM2G027023	uncharacterized protein LOC100279353 isoform 2
	GRMZM2G104262	cryptochrome 2
	GRMZM2G167651	golgin candidate 4 isoform X2
	GRMZM2G027023	uncharacterized protein LOC100279353 isoform 2
	GRMZM2G167651	golgin candidate 4 isoform X2
zma-miR166l-3p	*GRMZM2G028086*	Calcium-dependent protein kinase 7
	GRMZM2G144407	uncharacterized protein LOC100192720
	GRMZM5G813259	CRM-domain containing factor CFM3, chloroplastic/mitochondrial-like
zma-miR167c-3p_L-1	*GRMZM2G102475*	putative pinin_SDK_memA domain family protein isoform 2
	GRMZM2G153181	uncharacterized protein LOC100384046
	GRMZM2G179097	cyclin-dependent kinase G-2-like isoform X1
	GRMZM2G066449	LOW QUALITY PROTEIN: atherin
	XM_008675682.3	BSD domain-containing protein 1
	XM_008682762.2	putative pinin_SDK_memA domain family protein isoform 2
zma-miR167e-5p	*GRMZM2G003656*	RING finger protein 13 precursor
	GRMZM2G096622	uncharacterized protein LOC100193197 isoform X1
	GRMZM2G003656	RING finger protein 13 precursor
zma-MIR167j-p3 _2ss12AT20TA	*GRMZM2G160032*	putative transcription factor
zma-miR169o-3p _L-1R+1	*GRMZM2G104078*	putative NAC domain transcription factor superfamily protein
	GRMZM2G067225	allene-oxide synthase1
	GRMZM5G822947	uncharacterized protein LOC100276769
zma-miR169o-3p _L-1R+1	*GRMZM2G403797*	protein WHAT'S THIS FACTOR 1, chloroplastic
	Zm00001d035838	60S ribosomal protein L39-1
	GRMZM2G105400	uncharacterized LOC100281161
	GRMZM2G026447	uncharacterized protein LOC100281668
	GRMZM2G074017	ATPase inhibitor
	GRMZM2G031496	unknown
	Zm00001d018986	Protein WVD2-like 2

	GRMZM2G144387	unknown
	GRMZM2G072806	uncharacterized protein LOC100283266
	GRMZM5G890820	uncharacterized LOC100216726
	GRMZM2G107092	uncharacterized protein LOC107457590 isoform X1
	GRMZM2G082032	probable isoaspartyl peptidase/L-asparaginase 2
	GRMZM2G069254	uncharacterized LOC100275405
	GRMZM2G094581	E3 UFM1-protein ligase 1 homolog
	GRMZM2G389362	vacuolar-sorting receptor 1
	GRMZM2G117776	filaggrin-2
	GRMZM2G107092	uncharacterized protein LOC107457590 isoform X1
	GRMZM2G312110	auxin transport protein BIG
	GRMZM2G369991	60S ribosomal protein L10a
	GRMZM5G890820	uncharacterized LOC100216726
zma-miR169o-5p_R-1	*GRMZM2G115674*	uncharacterized protein LOC100277219
	GRMZM2G091964,	uncharacterized protein LOC100282719 isoform X1
	GRMZM2G141636	uncharacterized protein LOC100193940 isoform X1
	GRMZM5G829103	nuclear transcription factor Y subunit A-10 isoform X1
	GRMZM2G165488	uncharacterized LOC100193094
	GRMZM2G091964	uncharacterized protein LOC100282719 isoform X1
zma-MIR319a-p5	*GRMZM2G064390*	uncharacterized protein LOC100273603 isoform X1
	GRMZM2G044733	autophagy-related protein 13b
zma-MIR319b-p5	*GRMZM2G359038*	NA
	GRMZM2G359038,	uncharacterized protein LOC100272602 isoform X1
zma-MIR319d-p5	*GRMZM2G478417*	bZIP transcription factor TRAB1
zma-miR390a-5p	*GRMZM2G416701*	AP2-like ethylene-responsive transcription factor TOE3
	GRMZM2G042920	Transcription factor PIF4
	GRMZM2G134396	uncharacterized protein LOC100275036
zma-miR396a-5p	*GRMZM2G107092*	uncharacterized protein LOC107457590 isoform X1
zma-miR396c_L-1	*GRMZM2G055782*	MADS-box transcription factor 27 isoform X1
zma-miR396c_L-1	*GRMZM2G152908*	sucrose synthase 2 isoform X1
	GRMZM2G041223	putative growth-regulating factor 6
	GRMZM2G033612	uncharacterized protein LOC100101540
	GRMZM2G124566	growth-regulating factor
	GRMZM2G098594	putative growth-regulating factor 14
	GRMZM2G179941	stromal processing peptidase, chloroplastic
	GRMZM2G105335	putative growth-regulating factor 3
	GRMZM2G119359	putative growth-regulating factor 12
	GRMZM2G152908	sucrose synthase 2 isoform X1
zma-miR397a-5p	*GRMZM2G166659*	60S ribosomal protein L23a-like isoform X1 [Sorghum bicolor]
	GRMZM2G134806	hsc70-interacting protein
	GRMZM2G410152	uncharacterized protein LOC100286327
zma-MIR397b-p5	*GRMZM2G033230*	putative bZIP transcription factor superfamily protein
	GRMZM2G006119	corticosteroid 11-beta-dehydrogenase isozyme 1
	GRMZM2G120652	Pyridoxal 5'-phosphate synthase-like subunit PDX1.2

	GRMZM2G134806	hsc70-interacting protein
	Zm00001d038980	IAA19 - auxin-responsive Aux/IAA family member
	GRMZM5G834758	DNA-binding protein, partial
	GRMZM2G139349	40S ribosomal protein S25-1
	GRMZM5G867518	uncharacterized protein LOC100283674
	GRMZM2G397002	40S ribosomal protein S25-3
	GRMZM2G044348	NA
zma-miR398b-5p_R-1	*GRMZM2G080516*	AP2-EREBP transcription factor
	GRMZM2G131577	Transcription factor BTF3
	GRMZM2G141679	ethylene-responsive transcription factor RAP2-13-like
	GRMZM2G416701	AP2-like ethylene-responsive transcription factor TOE3
	GRMZM2G180452	
	GRMZM2G001444	unknown
	GRMZM2G177878	uncharacterized protein LOC100279545
	GRMZM2G131577	Transcription factor BTF3
	GRMZM2G338259	auxin response factor 10
	GRMZM2G409224	uncharacterized protein LOC100273824
	GRMZM2G001444	
zma-miR408a_L-1	*GRMZM2G180328*	putative NAC domain transcription factor superfamily protein
	GRMZM2G399072	AP2-like ethylene-responsive transcription factor BBM2 isoform X2
	GRMZM2G169688	transcription factor GTE8
	GRMZM2G093175	asparagine synthetase 2
zma-miR408a_L-1	*GRMZM2G002898*	*putative cytochrome P450 superfamily protein*
	GRMZM2G161245	*Malate dehydrogenase*
	GRMZM2G325118	*unknown*
	GRMZM2G134976	*uncharacterized protein LOC100275636*
	GRMZM2G478709	*pre-mRNA-splicing factor prp45*
	GRMZM2G488465	*uncharacterized protein LOC100381775*
	GRMZM2G077340	*hypothetical protein Zm00014a_040048*
zma-miR408a_L-1	*GRMZM2G336456*	*uncharacterized protein LOC103647431*
zma-miR444a_1ss12TC	*GRMZM2G399072*	*AP2-like ethylene-responsive transcription factor BBM2 isoform X2*
	GRMZM2G001024	*putative RING/U-box superfamily protein*
	GRMZM2G439201	*elongation factor 1-beta*
	GRMZM2G127139	*zeaxanthin epoxidase*
	GRMZM2G380377	*DRE-binding protein 4*
	GRMZM2G096764	*ATP-dependent zinc metalloprotease FTSH 4 mitochondrial*
	GRMZM2G064767	*unknown*
	GRMZM2G001024	*putative RING/U-box superfamily protein*
	GRMZM2G179325	*scarecrow-like protein 34*
	GRMZM2G127139	*zeaxanthin epoxidase*
zma-miR529-5p	*GRMZM2G146862*	*general transcription factor 3C polypeptide 3 isoform X1*
	GRMZM6G729818	*uncharacterized protein LOC100191463*
	GRMZM2G031501	*uncharacterized protein LOC100272713*
	GRMZM2G017804	*uncharacterized protein LOC100277723 precursor*

	GRMZM2G061734	*SBP-transcription factor 5*
	GRMZM2G074489	*probable protein phosphatase 2C 44*
	GRMZM2G061734	*SBP-transcription factor 5*
	GRMZM2G095782	*tRNA pseudouridine synthase*
zma-miR827-3p	*GRMZM2G003992*	*uncharacterized protein LOC100279277*
	GRMZM2G077531	*uncharacterized protein LOC100383043*
zma-miR827-5p_L+1	*GRMZM2G536825*	*NA*
zma-miR169o-5p_R-1	*GRMZM2G536825*	*L10-interacting MYB domain-containing protein isoform X2*
