# Supplementary material for: Identification of miRNAs Mediating Seed Storability of Maize during Germination Stage by High-Throughput Sequencing, Transcriptome and Degradome Sequencing
Source: Int J Mol Sci. 2022 Oct 15;23(20):12339. doi: 10.3390/ijms232012339 (PMC9604548; doi:10.3390/ijms232012339)
Supplement: Supplementary file 1 [file ijms-23-12339-s001.zip › Supplementary Table S4.pdf]

Supplementary Table S4 Putative miRNA and its corresponding target gene information

MiRNA	Transcript	Describtion
PC-3p-25167_195	*GRMZM5G803490*	uncharacterized protein LOC100284589
PC-3p-46024_102	*GRMZM2G104396*	Nuclear transcription factor Y subunit A-7
PC-3p-77755_56	*GRMZM2G025062*	AP2-EREBP transcription factor, partial
	Zm00001d019824	uncharacterized protein LOC100381531
	GRMZM2G058948	K(+) efflux antiporter 2, chloroplastic
PC-3p-87703_49	*GRMZM2G172529*	uncharacterized protein LOC100192543
	GRMZM2G133006	uncharacterized protein LOC100272795
PC-3p-91946_46	*GRMZM2G016602*	protein recA isoform X1
PC-5p-46450_101	*GRMZM2G009871*	putative TCP-1/cpn60 chaperonin family protein
	GRMZM2G009871	putative TCP-1/cpn60 chaperonin family protein
PC-5p-58549_78	*GRMZM2G421212*	GATA transcription factor 26
	GRMZM2G175425	uncharacterized protein LOC100275644
	GRMZM2G140150	Pyruvate dehydrogenase E1 component alpha subunit
	GRMZM2G151387	uncharacterized protein LOC100381870
	GRMZM2G147811	Protein kinase superfamily protein
PC-5p-58650_78	*GRMZM2G347043*	NAC1 transcription factor
	GRMZM2G014022	probable F-box protein At2g36090
PC-5p-71261_62	*GRMZM2G304712*	uncharacterized protein LOC100281303
	GRMZM2G153815	heat shock 70 kDa protein
PC-5p-80184_54	*GRMZM2G347043*	NAC1 transcription factor
	GRMZM2G120320	WRKY transcription factor WRKY71
	Zm00001d037605	GATA transcription factor 23
PC-5p-8044_611	*GRMZM2G006578*	putative bZIP transcription factor superfamily protein isoform X1
	GRMZM2G120320	WRKY transcription factor WRKY71
	GRMZM2G086573	AP2-like ethylene-responsive transcription factor AIL5
	GRMZM2G006578	putative bZIP transcription factor superfamily protein isoform X1
	GRMZM2G127548	BSD domain containing protein
