# Supplementary material for: Identification of miRNAs Mediating Seed Storability of Maize during Germination Stage by High-Throughput Sequencing, Transcriptome and Degradome Sequencing
Source: Int J Mol Sci. 2022 Oct 15;23(20):12339. doi: 10.3390/ijms232012339 (PMC9604548; doi:10.3390/ijms232012339)
Supplement: Supplementary file 1 [file ijms-23-12339-s001.zip › Supplementary Table S5.pdf]

Supplementary Table S5 GO enrichment circle of differentially expressed genes in comparison group AA156VSAA237

Index	GO_ID	GO_Category	B	S	Up_Num	Down_Num	Rich.Factor	-log10(Q)	hjust	angle	TB_Fill	Text_Colour
1	GO:0042939	Biological Process	23	16	11	5	0.6957	1.072896	0	-6	#FB9272	white
2	GO:0009741	Biological Process	55	32	21	11	0.5818	1.302728	0	-19	#FB9272	white
3	GO:0009969	Biological Process	48	27	14	13	0.5625	0.897727	0	-32	#FB9272	white
4	GO:0009832	Biological Process	94	48	31	17	0.5106	1.111182	0	-45	#FB9272	white
5	GO:0052696	Biological Process	205	104	28	76	0.5073	3.78599	0	-58	#F8684B	white
6	GO:0009813	Biological Process	237	112	31	81	0.4726	2.610072	0	-71	#F8684B	white
7	GO:0007169	Biological Process	202	90	58	32	0.4455	1.072896	0	-84	#F8684B	white
8	GO:0009409	Biological Process	322	134	68	66	0.4161	1.008329	0	85	#F8684B	white
9	GO:0006468	Biological Process	1022	387	224	163	0.3787	1.072896	0	71.66667	#F8684B	white
10	GO:0000795	Cellular Component	20	13	11	2	0.65	0.49545	0	58.33333	#FB9272	white
11	GO:0043231	Cellular Component	456	192	71	121	0.4211	1.943756	0	45	#F8684B	white
12	GO:0009505	Cellular Component	324	133	60	73	0.4105	0.812026	0	31.66667	#F8684B	white
13	GO:0046658	Cellular Component	267	109	46	63	0.4082	0.49545	0	18.33333	#F8684B	white
14	GO:0005768	Cellular Component	419	171	113	58	0.4081	1.072896	0	5	#F8684B	white
15	GO:0000139	Cellular Component	377	149	92	57	0.3952	0.49545	0	-6	#F8684B	white
16	GO:0005576	Cellular Component	1302	482	243	239	0.3702	1.008329	0	-19	#F8684B	white
17	GO:0005794	Cellular Component	1439	530	321	209	0.3683	1.021239	0	-32	#F8684B	white
18	GO:0005886	Cellular Component	4108	1464	858	606	0.3564	1.943756	0	-45	#F8684B	white
19	GO:0016021	Cellular Component	3605	1285	746	539	0.3564	1.537741	0	-58	#F8684B	white
20	GO:0042937	Molecular Function	23	16	11	5	0.6957	1.072896	0	-71	#FB9272	black
21	GO:0016705	Molecular Function	33	21	6	15	0.6364	1.072896	0	-84	#FB9272	black
22	GO:0008061	Molecular Function	44	26	5	21	0.5909	1.072896	0	85	#FB9272	black
23	GO:0016759	Molecular Function	49	28	22	6	0.5714	1.064647	0	71.66667	#FB9272	black

24	GO:0004568	Molecular Function	49	28	6	22	0.5714	1.064647	0	58.33333	#FB9272	black
25	GO:0080043	Molecular Function	180	93	25	68	0.5167	3.78599	0	45	#F8684B	black
26	GO:0080044	Molecular Function	180	93	25	68	0.5167	3.78599	0	31.66667	#F8684B	black
27	GO:0016757	Molecular Function	481	196	119	77	0.4075	1.218809	0	18.33333	#F8684B	black
28	GO:0004674	Molecular Function	1106	411	243	168	0.3716	0.814395	0	5	#F8684B	black
