# Supplementary material for: Identification of miRNAs Mediating Seed Storability of Maize during Germination Stage by High-Throughput Sequencing, Transcriptome and Degradome Sequencing
Source: Int J Mol Sci. 2022 Oct 15;23(20):12339. doi: 10.3390/ijms232012339 (PMC9604548; doi:10.3390/ijms232012339)
Supplement: Supplementary file 1 [file ijms-23-12339-s001.zip › Supplementary Table S6.pdf]

Supplementary Table S6 KEGG level histogram of differentially expressed genes in comparison group AA156VSAA237

Index	Pathway_ID	KEGG_Level_1	B	S	Up_Num	Down_Num	Rich.Factor	-log10(P)
1	zma04146	Cellular Processes	214	87	28	59	0.4065	2.183462
2	zma04144	Cellular Processes	494	170	101	69	0.3441	0.742772
3	zma04136	Cellular Processes	83	25	14	11	0.3012	0.147602
4	zma04145	Cellular Processes	211	63	34	29	0.2986	0.091641
5	zma02010	Environmental Information Processing	212	79	44	35	0.3726	1.120632
6	zma04075	Environmental Information Processing	842	291	142	149	0.3456	1.034113
7	zma04016	Environmental Information Processing	495	168	70	98	0.3394	0.608224
8	zma04070	Environmental Information Processing	212	71	39	32	0.3349	0.403461
9	zma03410	Genetic Information Processing	106	41	25	16	0.3868	0.990936
10	zma04141	Genetic Information Processing	570	200	118	82	0.3509	1.043236
11	zma00970	Genetic Information Processing	163	57	35	22	0.3497	0.571777
12	zma04122	Genetic Information Processing	23	8	1	7	0.3478	0.316842
13	zma04120	Genetic Information Processing	358	116	50	66	0.324	0.279984
14	zma03420	Genetic Information Processing	266	84	39	45	0.3158	0.19362
15	zma03440	Genetic Information Processing	279	87	44	43	0.3118	0.158218
16	zma03430	Genetic Information Processing	207	63	41	22	0.3043	0.122375
17	zma03030	Genetic Information Processing	238	69	41	28	0.2899	0.051406
18	zma03450	Genetic Information Processing	11	3	2	1	0.2727	0.127211
19	zma04130	Genetic Information Processing	69	17	7	10	0.2464	0.027737
20	zma01502	Human Diseases	2	1	1	0	0.5	0.264844
21	zma04933	Human Diseases	70	24	8	16	0.3429	0.383558
22	zma00073	Metabolism	66	34	19	15	0.5152	3.004621
23	zma00053	Metabolism	113	55	23	32	0.4867	3.639326

24	zma00520	Metabolism	320	142	74	68	0.4438	5.383796
25	zma00052	Metabolism	207	88	47	41	0.4251	2.858479
26	zma00500	Metabolism	446	178	81	97	0.3991	3.368156
27	zma04626	Organismal Systems	553	207	109	98	0.3743	2.205636
28	zma04712	Organismal Systems	214	57	29	28	0.2664	0.011944
