# Supplementary material for: Identification of miRNAs Mediating Seed Storability of Maize during Germination Stage by High-Throughput Sequencing, Transcriptome and Degradome Sequencing
Source: Int J Mol Sci. 2022 Oct 15;23(20):12339. doi: 10.3390/ijms232012339 (PMC9604548; doi:10.3390/ijms232012339)
Supplement: Supplementary file 1 [file ijms-23-12339-s001.zip › Supplementary Table S7.pdf]

Supplementary Table S7 GO terms for miRNA-mRNA

miRNA-mRNA-GO		
A:miR169o-5p-GRMZM2G165488- GO:0016602\ GO:0009793\ GO:0006355-DGEs		
GO	Gene	Description
GO:0016602	GRMZM2G091964	uncharacterized protein
GO:0016602	GRMZM2G104396	Nuclear transcription factor Y subunit A-7
GO:0016602	GRMZM2G165488	uncharacterized protein
GO:0016602	GRMZM5G829103	nuclear transcription factor Y subunit A-10 isoform X1
GO:0009793	GRMZM2G091964	uncharacterized protein
GO:0009793	GRMZM2G134396	Nuclear transcription factor Y subunit A-7
GO:0009793	GRMZM2G347043	NAC1 transcription factor
GO:0009793	GRMZM5G813259	CRM-domain containing factor CFM3, chloroplastic/mitochondrial-like
GO:0009793	GRMZM2G002898	putative cytochrome P450 superfamily protein
GO:0009793	GRMZM2G161245	Malate dehydrogenase
GO:0006355	GRMZM2G033230	putative bZIP transcription factor superfamily protein
GO:0006355	GRMZM2G179325	scarecrow-like protein 34
GO:0006355	GRMZM2G416701	AP2-like ethylene-responsive transcription factor TOE3
GO:0006355	GRMZM2G033612	uncharacterized protein
GO:0006355	GRMZM2G165488	uncharacterized protein
GO:0006355	GRMZM2G380377	DRE-binding protein 4
GO:0006355	GRMZM2G478417	bZIP transcription factor TRAB1
GO:0006355	GRMZM2G399072	AP2-like ethylene-responsive transcription factor BBM2 isoform X2
GO:0006355	GRMZM2G416701	AP2-like ethylene-responsive transcription factor TOE3
GO:0006355	GRMZM2G061734	SBP-transcription factor 5
GO:0006355	GRMZM2G180328	putative NAC domain transcription factor superfamily protein
GO:0006355	mbd109	unknown
GO:0006355	GRMZM2G104396	Nuclear transcription factor Y subunit A-7
GO:0006355	GRMZM5G829103	nuclear transcription factor Y subunit A-10 isoform X1
GO:0006355	GRMZM2G086573	AP2-like ethylene-responsive transcription factor AIL5
GO:0006355	GRMZM2G131577	Transcription factor BTF3
GO:0006355	GRMZM2G119865	ethylene-responsive transcription factor RAP2-9
GO:0006355	GRMZM2G061734	SBP-transcription factor 5
GO:0006355	GRMZM2G141679	ethylene-responsive transcription factor RAP2-13-like
GO:0006355	GRMZM2G091964	uncharacterized protein LOC100282719 isoform X1
GO:0006355	GRMZM2G399072	AP2-like ethylene-responsive transcription factor BBM2 isoform X2
GO:0006355	GRMZM2G488465	uncharacterized protein
GO:0006355	GRMZM2G025062	AP2-EREBP transcription factor, partial

GO:0006355	GRMZM2G104078	putative NAC domain transcription factor superfamily protein
GO:0006355	GRMZM2G133568	MADS-box transcription factor 27 isoform X1
GO:0006355	GRMZM2G124566	growth-regulating factor
GO:0006355	GRMZM2G409224	uncharacterized protein
B:zma-miR390a-5p-GRMZM2G134396\ GO:0009737\ GO:0009793\ GO:0003876-DEGs		
GO	Gene	Description
GO:0009737	GRMZM2G478709	pre-mRNA-splicing factor prp45
GO:0009737	GRMZM2G338259	auxin response factor 10
GO:0009737	GRMZM5G813259	CRM-domain containing factor CFM3, chloroplastic/mitochondrial-like
GO:0009737	GRMZM2G006119	corticosteroid 11-beta-dehydrogenase isozyme 1
GO:0009737	GRMZM2G134396	WRKY transcription factor, partial
GO:0009793	GRMZM2G091964	uncharacterized protein LOC100282719 isoform X1
GO:0009793	GRMZM2G134396	WRKY transcription factor, partial
GO:0009793	GRMZM2G347043	NAC1 transcription factor
GO:0009793	GRMZM5G813259	CRM-domain containing factor CFM3, chloroplastic/mitochondrial-like
GO:0009793	GRMZM2G002898	putative cytochrome P450 superfamily protein
GO:0009793	GRMZM2G161245	Malate dehydrogenase
GO:0003876	GRMZM2G134396	WRKY transcription factor, partial
C:zma-miR396c_L-1-GRMZM2G098594\ GO:0008757\ GO:0032259\ GO:0009739\ GO:0030912-DEGs		
GO	Gene	Description
GO:0008757	GRMZM2G041223	putative growth-regulating factor 6
GO:0008757	GRMZM2G119359	putative growth-regulating factor 12
GO:0008757	GRMZM2G098594	putative growth-regulating factor 14
GO:0032259	GRMZM2G041223	putative growth-regulating factor 6
GO:0032259	GRMZM2G119359	putative growth-regulating factor 12
GO:0032259	GRMZM2G098594	putative growth-regulating factor 14
GO:0032259	GRMZM2G172529	uncharacterized protein
GO:0009739	GRMZM2G133568	MADS-box transcription factor 27 isoform X1
GO:0030912	GRMZM2G133568	MADS-box transcription factor 27 isoform X1
D:zma-miR396c_L-1-GRMZM2G105335\ GO:0009739\ GO:0030912-DEGs		
GO	Gene	Description
GO:0009739	GRMZM2G105335	putative growth-regulating factor 3
GO:0030912	GRMZM2G105335	putative growth-regulating factor 3
E:zma-miR396c_L-1-GRMZM2G133568\ GO:0000977\ GO:0007275-DEGs		
GO	Gene	Description
GO:0000977	GRMZM2G421212	GATA transcription factor 26
GO:0000977	GRMZM2G133568	MADS-box transcription factor 27 isoform X1
GO:0000977	GATA23	GATA transcription factor 23
GO:0007275	GRMZM2G399072	AP2-like ethylene-responsive transcription factor BBM2 isoform X2
GO:0007275	GRMZM2G347043	NAC1 transcription factor

GO:0007275	GRMZM2G086573	AP2-like ethylene-responsive transcription factor AIL5
GO:0007275	GRMZM2G104078	putative NAC domain transcription factor superfamily protein
GO:0007275	GRMZM2G180328	putative NAC domain transcription factor superfamily protein
GO:0007275	GRMZM2G177878	uncharacterized protein
GO:0007275	GRMZM2G040762	uncharacterized protein

F:zma-miR397b-p5-GRMZM2G033230\ GO:0006355\ GO：0006970\ GO：0009409\ GO：0009651-DEGs

GO	Gene	Description
GO:0006355	GRMZM2G033230	putative bZIP transcription factor superfamily protein
GO:0006355	GRMZM2G179325	scarecrow-like protein 34
GO:0006355	GRMZM2G416701	AP2-like ethylene-responsive transcription factor TOE3
GO:0006355	GRMZM2G033612	uncharacterized protein
GO:0006355	GRMZM2G165488	uncharacterized protein
GO:0006355	GRMZM2G380377	DRE-binding protein 4
GO:0006355	GRMZM2G478417	bZIP transcription factor TRAB1
GO:0006355	GRMZM2G399072	AP2-like ethylene-responsive transcription factor BBM2 isoform X2
GO:0006355	GRMZM2G416701	AP2-like ethylene-responsive transcription factor TOE3
GO:0006355	GRMZM2G061734	SBP-transcription factor 5
GO:0006355	GRMZM2G180328	putative NAC domain transcription factor superfamily protein
GO:0006355	mbd109	unknown
GO:0006355	GRMZM2G104396	Nuclear transcription factor Y subunit A-7
GO:0006355	GRMZM5G829103	nuclear transcription factor Y subunit A-10 isoform X1
GO:0006355	GRMZM2G086573	AP2-like ethylene-responsive transcription factor AIL5
GO:0006355	GRMZM2G131577	Transcription factor BTF3
GO:0006355	GRMZM2G119865	ethylene-responsive transcription factor RAP2-9
GO:0006355	GRMZM2G061734	SBP-transcription factor 5
GO:0006355	GRMZM2G141679	ethylene-responsive transcription factor RAP2-13-like
GO:0006355	GRMZM2G091964	uncharacterized protein LOC100282719 isoform X1
GO:0006355	GRMZM2G399072	AP2-like ethylene-responsive transcription factor BBM2 isoform X2
GO:0006355	GRMZM2G488465	uncharacterized protein LOC100381775
GO:0006355	GRMZM2G025062	AP2-EREBP transcription factor, partial
GO:0006355	GRMZM2G104078	putative NAC domain transcription factor superfamily protein
GO:0006355	GRMZM2G133568	MADS-box transcription factor 27 isoform X1
GO:0006355	GRMZM2G124566	growth-regulating factor
GO:0006355	GRMZM2G409224	uncharacterized protein LOC100273824
GO：0006970	GRMZM2G033230	putative bZIP transcription factor superfamily protein
GO：0006970	GRMZM2G141679	ethylene-responsive transcription factor RAP2-13-like
GO：0009409	GRMZM2G380377	DRE-binding protein 4
GO：0009409	GRMZM2G161245	Malate dehydrogenase
GO：0009409	GRMZM2G141679	ethylene-responsive transcription factor RAP2-13-like
GO：0009409	GRMZM2G033230	putative bZIP transcription factor superfamily protein

GO：0009651	GRMZM2G141679	ethylene-responsive transcription factor RAP2-13-like
GO：0009651	GRMZM6G729818	uncharacterized protein LOC100191463
GO：0009651	GRMZM2G153815	heat shock 70 kDa protein
GO：0009651	GRMZM2G033230	putative bZIP transcription factor superfamily protein
GO：0009651	GRMZM2G131577	Transcription factor BTF3
GO：0009651	GRMZM2G120652	Pyridoxal 5'-phosphate synthase-like subunit PDX1.2
GO：0009651	GRMZM2G478709	pre-mRNA-splicing factor prp45
H:miR444a-GRMZM2G399072- GO：0007275\ GO：0009873\ GO：1905392\ GO:0005524-DEGs		
GO	Gene	Description
GO：0007275	GRMZM2G399072	AP2-like ethylene-responsive transcription factor BBM2 isoform X2
GO：0007275	GRMZM2G347043	NAC1 transcription factor
GO：0007275	GRMZM2G086573	AP2-like ethylene-responsive transcription factor AIL5
GO：0007275	GRMZM2G133568	MADS-box transcription factor 27 isoform X1
GO：0007275	GRMZM2G104078	putative NAC domain transcription factor superfamily protein
GO：0007275	GRMZM2G180328	putative NAC domain transcription factor superfamily protein
GO：0007275	GRMZM2G177878	uncharacterized protein
GO：0007275	GRMZM2G040762	uncharacterized protein
GO：0009873	GRMZM2G399072	AP2-like ethylene-responsive transcription factor BBM2 isoform X2
GO：0009873	GRMZM2G119865	ethylene-responsive transcription factor RAP2-9
GO：0009873	GRMZM2G086573	ponsive transcription factor AIL5
GO：0009873	GRMZM2G141679	ethylene-responsive transcription factor RAP2-13-like
GO：0009873	GRMZM2G025062	AP2-EREBP transcription factor, partial
GO：1905392	GRMZM2G399072	AP2-like ethylene-responsive transcription factor BBM2 isoform X2
GO：1905392	GRMZM2G086573	AP2-like ethylene-responsive transcription factor AIL5
GO:0005524	GRMZM2G104262	cryptochrome 2
GO:0005524	GRMZM2G359038	uncharacterized protein
GO:0005524	GRMZM2G124566	growth-regulating factor
GO:0005524	GRMZM5G890820	uncharacterized protein

Note: (A) zma-miR169o-5p_ R-1-*GRMZM2G165488* and related GO terms "GO: 0006355, GO: 0009793 and GO: 0016602" corresponding DEGs. (B) zma-miR390a-5p-*GRMZM2G134396* and related GO terms " GO : 0009737, GO:0009793 , GO:0003876" and corresponding DEGs. (C) zma-miR396c_ L-1- *GRMZM2G098594* and related GO terms " GO: 008757, GO-0032259 , GO: 0009739 , GO: 0030912 and other corresponding DEGs. (D) zma-miR396c_ L-1- *GRMZM2G105335* and related GO terms "GO: 0009739, GO: 0030912 and other corresponding DEGs. (E) zma-miR396c_ L-1- *GRMZM133568* and related GO terms " GO: 0000977, GO: 007275" and other corresponding DEGs. (F) zma-miR397b-p5-*GRMZM2G033230* and related GO terms"GO:0006355，GO：0006970，GO：0009409 and GO：0009651 "corresponding DEGs. (G) zma-miR444a_1ss12TC-*GRMZM2G399072* and related GO terms"GO ： 0007275,GO ： 0009873 and GO ： 1905392"corresponding DEGs. (H) novel-miR4-*GRMZM2G009871* and related GO terms"GO:0046686 and GO:0005524" corresponding DEGs.
