# Supplementary material for: Identification of miRNAs Mediating Seed Storability of Maize during Germination Stage by High-Throughput Sequencing, Transcriptome and Degradome Sequencing
Source: Int J Mol Sci. 2022 Oct 15;23(20):12339. doi: 10.3390/ijms232012339 (PMC9604548; doi:10.3390/ijms232012339)
Supplement: Supplementary file 1 [file ijms-23-12339-s001.zip › Supplementary Table S8.pdf]

Supplementary Table S8 Sorting of sequencing data

Group	Definition
group1a	Reads can be compared to the known pre miRNAs of miRBase, and the precursors can be further compared to the genome
group1b	Reads can be compared to the pre miRNAs of selected species known to miRBase, and the precursors can be further compared to the genome
group2a	Reads can be compared to the precursors (pre miRNAs) of miRBase selected species. Precursors cannot be further compared to the genome of this species, but reads can be compared to the genome. Extended genome sequence can form hairpin structure meeting 11 principles
group2b	Reads can be compared to the precursors (pre miRNAs) of miRBase selected species. Precursors cannot be further compared to the genome of this species, but reads can be compared to the genome. The extended genome sequence cannot form a hairpin structure meeting the 11 principles
group3a	Reads can be compared to the precursors (pre miRNAs) of miRBase selected species, and the precursors cannot be further compared to the genome, nor can reads be compared to the genome. The number of Reads on the comparison in the cluster, the number of copies ＞ 1, and the representative score of Reads ＞ 260. There is no mismatch
group4a (novel miRNAs)	Reads cannot be compared to the precursors (pre miRNAs) of miRBase selected species. Reads can be compared to the genome. Extended genome sequence can form hairpin structure meeting 11 principles
