# Supplementary material for: Identification of miRNAs Mediating Seed Storability of Maize during Germination Stage by High-Throughput Sequencing, Transcriptome and Degradome Sequencing
Source: Int J Mol Sci. 2022 Oct 15;23(20):12339. doi: 10.3390/ijms232012339 (PMC9604548; doi:10.3390/ijms232012339)
Supplement: Supplementary file 1 [file ijms-23-12339-s001.zip › Supplementary Table S9.pdf]

Supplementary Table S9 Primer sequences

miRNA /Gene name	Primers	Primer sequence(5'-3')
U6	F	GGAGGCAGAGGCATTGGA
	R	CCCACCTTTGTTACCACCTTTG
zma-miR169o-5p_R-1	RT primer	GTCGTATCCAGTGCAGGGTCCGAGGTAT TCGCACTGGATACGACAGGCAA
	Forward primer	CGCTGCATTAGCCAAGAATGA
zma-miR390a-5p	RT primer	GTCGTATCCAGTGCAGGGTCCGAGGTAT TCGCACTGGATACGACGGCGCT
	Forward primer	AATTGTACAAGCTCAGGAGGGA
zma-miR396c_L-1	RT primer	GTCGTATCCAGTGCAGGGTCCGAGGTAT TCGCACTGGATACGACCAGTTC
	Forward primer	AACGGCTCCACAGGCTTTC
zma-miR444a_1ss12TC	RT primer	GTCGTATCCAGTGCAGGGTCCGAGGTAT TCGCACTGGATACGACAAGCTT
	Forward primer	AATTGTACTGCAGTTGTTGCCT
zma-miR397b-p5	RT primer	GTCGTATCCAGTGCAGGGTCCGAGGTATTCGCACT GGATACGACGCTCATC
	Forward primer	AAGCACTTGAGCGCAGCGTT
PC-5p-46450_101 （novel-miR）	RT primer	GTCGTATCCAGTGCAGGGTCCGAGGTATTCGCACT GGATACGACGCAATGA
	Forward primer	CGCCGGTCCAAGTCAATGCA
General reverse primer		CAGTGCAGGGTCCGAGGTAT
β-actin	β-actin-F	CATCCACGAGACCACCTACAA
	β-actin-R	CGAGCCACCAATCCAAACA
GRMZM2G165488	*GRMZM2G165488-F*	CCGATGGCTGGTTGTCTGTAGG
	GRMZM2G165488-R	ACCCAAGCACCCTGGAATTTCA
GRMZM2G134396	*GRMZM2G134396-F*	CGACTATCATCGGAACCC
	GRMZM2G134396-R	TCCAAAGTGAAGCAGCAA
GRMZM2G105335	*GRMZM2G105335-F*	CACCAGAAGGGGGACAAGG
	GRMZM2G105335-R	CCACCGTTACGGAAGCG
GRMZM2G098594	*GRMZM2G098594-F*	AGCGTCATCGGGGAACAA
	GRMZM2G098594-R	TGGGAGTGGGACTGGGAGTA
GRMZM2G399072	*GRMZM2G399072*-F	GGGTCGCTGGCAATAAGGA
	GRMZM2G399072-R	AAGTTGGTCACCGCGTTGAG
GRMZM2G133568	*GRMZM2G133568*-F	TGATGGGAGAGGATCTTTCTGG
	GRMZM2G133568-R	CCTTTCGATTCAATTCGTGGA
GRMZM2G033230	*GRMZM2G033230*-F	ATGGACGATGGAGCTGACCT
	GRMZM2G033230-R	CGCTGCTGAAGAACTCGTCG
GRMZM2G009871	*GRMZM2G009871-F*	GCTCATCCACGACGACAAGG
	GRMZM2G009871-R	CAGTAGTTGTCCCATCGCCG
GRMZM2G165488	*GRMZM2G165488-GNSP*	CAGCAGCAGCAGCAGCAGCAGCAGCGACCA
	GRMZM2G165488-GSP	CAGCAGCAGCGACCATAATGGAGGCGCCAA

GRMZM2G399072	*GRMZM2G399072-GNSP*	TCATTGAAGAATAACATGCATG
	GRMZM2G399072-GSP	TGCATGCATGTGGAACCATTAC
